# Cervical Dystonia Resolving After the Onset of Parkinson’s Disease

**DOI:** 10.7759/cureus.12595

**Published:** 2021-01-09

**Authors:** Juan Fernando Ortiz, Sagari Betté, Stuart Isaacson, Maria A Hernandez, Jazmin Carolina Cozar

**Affiliations:** 1 Neurology, California Institute of Behavioral Neurosciences & Psychology, Fairfield, USA; 2 Neurology, Universidad San Francisco de Quito, Quito, ECU; 3 Movement Disorders Specialist, Parkinson's Disease and Movement Disorders Center of Boca Raton, Boca Raton, USA; 4 Neurology, Parkinson's Disease and Movement Disorder Center of Boca Raton, Boca Raton, USA; 5 Department of Neurology, Universidad Central de Venezuela, Caracas, VEN; 6 Medicine, Universidad de las Americas, Quito, ECU; 7 Family Medicine, Open Door Family, Portchester, USA

**Keywords:** parkinson' s disease, cervical dystonia

## Abstract

Dystonia can occur in Parkinson’s disease (PD). Dystonia usually presents during the course of the disease or as a side effect of medication. The development of cervical dystonia (CD) before the onset of PD is uncommon but has been described. Complete resolution of CD has not been described to date. We demonstrate a 71-year-old man with a four-year history of CD, who presented with prodromal PD symptoms. Three years later, he was diagnosed with PD. Shortly after initiating treatment for PD, his cervical dystonia started to improve, and eventually, he had complete resolution of CD approximately after two years of PD treatment. In conclusion, the pathological basis of this association is not well understood, but it is important to note that the globus pallidus internus (GPi) plays an important role in connecting these two disorders at a pathological level and as a target for surgery. Increased activity in the GPi may cause resolution of the CD by decreasing unintentional thalamocortical activity.

## Introduction

Parkinson's disease (PD) is the most common movement disorder, characterized by bradykinesia, resting tremor, autonomic dysfunction, and rigidity. PD and dystonia have been closely linked. Dystonia has been reported in at least 30% of cases, usually after initiating treatment with levodopa (L-Dopa) or another dopamine agonist [1]. Dystonia is very atypical in untreated patients and more commonly occurs as a complication of PD treatment. The dystonia in untreated patients can occur in two settings: the "off period of L-Dopa", and the peak dose period. The most common dystonia site for the "off period" is feet, and the most common site of the peak dose period is the face and the neck. "Off period" dystonia is usually relieved by dopamine agonists [2]. Dystonia has been reported as an initial symptom in 2.4% of patients. For patients below 40 years, it was in 14% of reported cases [3]. Cervical dystonia (CD) is less commonly associated with PD than other dystonia types like blepharospasm, limb dystonia, or camptocormia [1].

CD is a movement disorder characterized by abnormal muscle contraction, which involves involuntary twisting and neck movement [1]. The disease's pathophysiology remains mostly unclear. However, it has been proposed that increased excitability of the motor cortex due to low firing rates in the globus pallidus internus (GPi) decreases the inhibition of thalamic activity, leading to involuntary contractions of the agonist and antagonist muscles [4]. We present a patient with the onset of CD before diagnosing PD, who developed a complete resolution of his dystonia after two years of PD treatment. CD has been described in several anecdotal reports as a presenting symptom before the onset of PD. In these anecdotal reports, CD persisted after the emergence of PD. Our patient had complete resolution of CD after the beginning of L-Dopa. We conducted an overview of the literature of these reported patients and discussed a possible mechanism that might explain the improvement of CD after the diagnosis of PD to establish a better understanding of this association.

## Case presentation

A 71-year-old man presented to our movement disorder clinic for concerning symptoms. Before presenting to the clinic, he was diagnosed with CD four years ago. He mentioned that he had progressive tremor and rigidity of the head. The rigidity was not new but has gotten worse. However, the tremor in the head was new and appeared approximately six months ago. The patient described that his head moved to the right unintentionally, and he always needed to stop it by putting his left hand on his chin, which was very annoying to him. He mentioned that this happened more commonly during the day while he was reading or eating dinner. Additionally, he described that in the last year he started to developed daytime sleepiness, overnight awakenings, forgetfulness, difficulty concentrating, occasional shoulder pain, and loss of smell.

On initial examination, the first mini-mental exam (MMSE) was 30/30. Cranial nerves were intact except for the subjective reduced smell. The superficial sensation was normal. The motor exam showed reflexes 2+. The head's tremor was noted while the patient was sitting and looking straight, and limitations of the cervical range of motion, including flexion, rotation, and extension. The gait was normal, without exhibiting any balance issues. The muscle tone was normal except in the neck, which was clearly increased. Motor strength was 5/5 in all four extremities. Upon palpation on the neck, there was tenderness of sternocleidomastoid and scale muscles but without inflammation signs. The patient was treated with botulinum toxin A injections and continued the same treatment every six months.

His family history was unremarkable. In particular, he had no family history of movement disorders or any neurological disorder. Personal medical history included hypotension, mild bilateral neurosensorial hearing loss, and benign paroxysmal positional vertigo (BPPV). The patient had a cholecystectomy 20 years ago due to gallstones.

The patient continued with routine visits to the office every six months. Three years after the first visit, the patient started to have problems with his balance and also described trouble getting out of bed in the morning due to a feeling that his whole body was "frozen." The patient reported difficulty negotiating curbs and demonstrated difficulty with multitasking while walking. The patient said that he played golf about four times per week without problem except walking on the grass and stabilizing himself after getting out of the golf cart. The patient did not report any major falls. He also mentioned falling when rising suddenly from a sitting position. He continued having intermittent hypotension readings upon his general practitioner's examination and orthostatic hypotension episodes when examined in the movement disorder clinic.

Upon examination, he continued having the same CD signs, with increased rigidity and tenderness of the neck, but the neck's tremor was improving. The muscle tone was increased, slightly more on the right side of the body. The sensation was normal. When walking, he had decreased swinging of the arms and difficulty turning back. He exhibited decreased amplitude in the upper extremities' movements (bradykinesia), increasing rigidity in the upper and lower extremities. He denied having hallucinations or memory problems. We performed an MRI to rule out any structural damage. The MRI was completely normal. The symptoms and signs of the patient make us diagnosed the patient with PD.

Initial genetic testing for glucocerebrosidase (GBA) and leucine-rich repeat kinase-2 (LARRK-2) genes was negative, and the extended gene panel, including the parkin gene, is pending. We performed these tests in the movement disorder clinic for research purposes and established a patient's genetic database. We acknowledge that these genetic tests are not routinely performed or necessary for diagnosing PD. The patient began treatment with levodopa-carbidopa. Over the next few days, the patient reported an initial strong response and improved his bradykinesia symptoms. The shaking in the head also improved.

Remarkably, months later, the patient reported that the symptoms associated with his CD start to improve dramatically. One year later, after initiating the treatment with levodopa-carbidopa, the patient reported no longer having CD symptoms. Physical exam was also negative for CD signs. Today, the patient is 86 years old and no longer requires botulinum injections. However, his PD symptoms have gotten worse. He has rigidity in the upper and lower extremities, decreased speed and amplitude in his movements, and decreased motor strength of 4/5 in the upper extremities and lower extremities. The rigidity appears to be worst on the right side of his body. He has fallen two times in the last year, luckily without any fractures. The patient is retired and still functional in his daily activities. However, his finances are managed by his wife. The spouse sleeps in a different bed because of the patient's constant movement while sleeping, which is related to his rapid eye movement (REM) sleep disorder. His symptoms are managed today with a long-acting L-Dopa 5 and a short-acting L-Dopa inhaler used for his "off episodes".

## Discussion

CD presenting before PD has been briefly described in the literature. Papapetropoulos et al. reported three cases of CD preceding PD. Two patients were female, and one patient was male. The onset of PD after CD was one, three, and 12 years in these patients. All patients were treated with botox toxin A (BTX-A) injections for four to five months for their dystonic symptoms, without interruption after PD diagnosis. The patients presented an improvement of 90-95% of dystonic symptoms with the BTX-A treatment. L-Dopa did not have any effect on two patients, while another patient had "off period"-related oromandibular dystonia [3].

Another report by LeWitt et al. studied ten patients presenting with typical parkinsonian symptoms associated with dystonic features. One of these patients developed CD four years before Parkinson's symptoms were noted. This patient was 25 years old when his CD started. His Parkinson's symptoms improved with the administration of L-dopa. However, there was no improvement in his dystonic symptoms. Interestingly, when bromocriptine and lisuride were added, there was an increase in CD's symptoms [5].

Finally, Katchen and Duvoisin documented another case of PD following CD in a 49-year-old man. The man presented with mild levoscoliosis and torticollis with hyperextension of the trunk. Two years later, the patient was diagnosed with PD. The patient began treatment for PD with L-Dopa and bromocriptine, which worsened his dystonic features and did not change his Parkinson's symptoms. Interestingly, there was an improvement in 30% of both dystonia and parkinsonian features with the administration of trihexyphenidyl, an anticholinergic [6]. Table 1 presents patients with a summary of patients we found in the literature, presenting with CD before PD onset [3, 5, 6].

**Table 1:** Clinical features of patients with cervical dystonia as presenting symptom of Parkinson disease CD - cervical dystonia; L-Dopa - levodopa; PD - Parkinson's disease

Author, source	Gender	Age onset of CD	Age onset of PD	Features of CD	Features of CD after being diagnosed with PD	Features of PD
Our case	M	67	74	There was an involuntary movement of the neck to the right, rigidity in the neck, and dystonic tremor in the neck as well.	L-Dopa improved dystonic symptoms. After initiating treatment for PD, the CD's symptoms improved until they reached a point of a year after diagnosed with PD, in which the symptoms of his CD disappeared.	He had daytime sleepiness, overnight awakening, and anosmia. Later, he developed bilateral rigidity, bradykinesia, and trouble with ambulation. At age 86, his PD features became more prominent. He is taking long-acting and short-acting L-Dopa.
Papapetropoulos et al. [3]	F	51	54	There was an involuntary rotation of the neck to the left. A year later, she developed a dystonic tremor in the neck.	After starting L-Dopa, the CD symptoms did not improve. The condition has not progressed, and the patient’s illness remained stable. She managed her symptoms with botox injections.	Bradykinesia (L > R), resting tremor (L > R), rigidity of upper left extremity and depression. At age 54, the patient started L-Dopa. At age 56, amantadine was incorporated into the current regimen because her Parkinson’s symptoms deteriorated.
Papapetropoulos et al. [3]	M	54	55	The patient was feeling that his neck was pulling back to the right. The patient developed lateral retro colitis and laterolocollis.	Around age 60, her condition stopped progressing and his CD remains stable. L-dopa did not have an effect on his CD’s symptoms at first. He developed oromandibular dystonia related to L dopa “off period”. He manages his symptoms with botox injections.	Pronounced gait difficulties, bradykinesia (upper extremities > lower extremities), hypomimia, right hand resting tremor. After six years, the Parkinson’s symptoms progressed, so there was a necessity to put him on additional medications. He takes L-dopa, pergolide, entacapone, and selegiline.
Papapetropoulos et al. [3]	F	50	62	She started having involuntary neck movements to the right and head tremors. The symptoms remained mild. No treatment was required.	She develops intermittent torticollis and laterocollis to the right and deterioration of her dystonic head tremor. The dystonic symptoms were not influenced by anti-parkinsonian medications. The patient controls her symptoms with botox injections and clonazepam.	She had a typical resting tremor of her right hand and difficulties with ambulation. She also had rigidity in the right upper limb and neck. Two years after the diagnosis of PD, she is receiving L-Dopa, amantadine, selegiline, and ropinirole.
Stoessl et al. [4]	M	25	29	Unilateral torticollis with neck pain.	There was significant relief of torticollis after initiating treatment with Parkinson’s medication.	Bilateral Parkinson’s signs.
LeWitt et al. [5]	M	49	51	Involuntary closing of the eyes. A year later, he started having dystonia in his back. The patient also developed torticollis	Treatment with L-Dopa and bromocriptine worsened the dystonia without improving the Parkinson’s symptoms. Anticholinergics improve both dystonic and Parkinson’s symptoms. Over the course of four years, the dystonic features have diminished.	Mild bradykinesia, resting tremor, and increased tone in the right upper extremity. Over the course of four years, the manifestations of PD have become more prominent.

**Figure 1:**
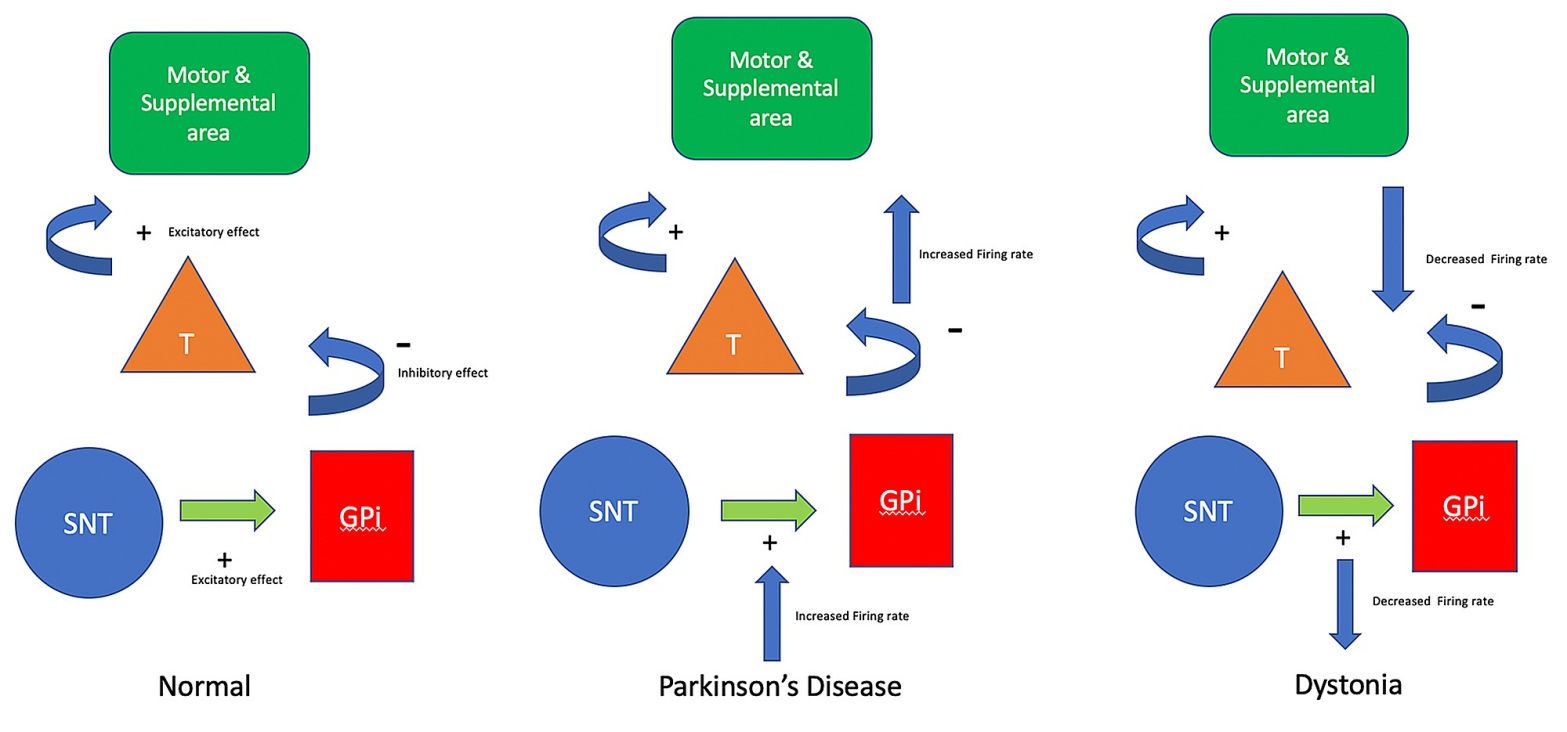
Motor circuit of Parkinson's disease and dystonia The SNT has a stimulatory effect on the GPi. When the GPi is stimulated, an inhibitory response of the GPi to the thalamus is produced. In Parkinson's disease, the firing rates of SNT and GPi are increased, leading to inhibition of the thalamus and less signaling to the motor and premotor cortex. Meanwhile, in dystonia, the firing rates of the SNT and GPi are decreased. In cervical dystonia, the thalamus is disinhibited, and more stimulation to the motor cortex is elicited. SNT - subthalamic nucleus; GPi - globus pallidus internus; T - thalamus

The characteristics of our patient differed from other patients in which CD preceded PD. The other patients were younger, between 25 and 51 when CD was diagnosed, and they were between 29 and 55 at the time PD was diagnosed [3, 5, 6]. Our patient was 67 years old when his CD started and 78 when his PD symptoms began. The increased subcortical degeneration with aging could explain the different outcomes [7]. The co-occurrence of PD and dystonia suggests a connection between these two diseases. PD manifests initially by losing dopamine in the substantia nigra and the dorsal striatum [8]. The pathophysiology of dystonia is complex and mostly unknown. However, it has been found that the decreased output of GPi to the thalamus is leading to decreased inhibition of the supplementary and premotor areas [9]. There is also decreased output of the subthalamus/subthalamic nucleus (STN) to the GPi. When the GPi is less stimulated by the STN, more movement is produced because of less inhibition of the GPi over the thalamus [10]. Meanwhile, in PD, the firing rates in the GPi and subthalamus STN increased, leading to inhibition of the thalamus and producing less excitatory output to the premotor and motor areas [11]. Figure 1 highlights the difference in the motor circuits in PD and CD [9-11].

Furthermore, the GPi plays a role in surgical targeting in deep brain stimulation for both conditions. Stimulation of the GPi in a patient with CD produced PD, showing a connection between these two disorders [11]. Firing rates in the GPi in CD are lower compared to patients with PD. Suppose a patient presents with CD before the onset of PD. In that case, we could hypothesize that the low firing rates in GPi would normalize or increase because of PD pathophysiologic basis, where firing rates in GPi are increased [11]. Patients with CD could, in theory, reverse or alleviate their symptoms if their firing rates in GPi increase. CD and PD's pathological bases in the GPi may explain how PD's symptoms develop, and treatment of the disease can lessen CD symptoms. Our study suggests a conjecture which merits further exploration to have a better understanding of these conditions.

## Conclusions

The pathological basis of this association between CD and PD is not well understood, but it is important to note that the GPi plays an important role in connecting these two disorders at a pathological level and as a target for surgery. Increased activity in the GPi may cause resolution of the cervical dystonia by decreasing unintentional thalamocortical activity. Patients with CD could theoretically reverse or ameliorate their symptoms if their firing rates in GPi increase. The pathological bases of CD and PD in the GPi may explain how the development and treatment of PD can ameliorate the symptoms of CD. The biggest difference between our patient and other reported patients was that our patient improved after the treatment with L-dopa. The other reported patients were commonly around 50 years of age, whereas our patient was 67 years old when his CD started and 74 years old when his PD symptoms began. The increased subcortical degeneration with aging could explain the different outcomes.
